# Impacts of Changing Winters on Lake Ecosystems Will Increase With Latitude

**DOI:** 10.1111/ele.70200

**Published:** 2025-08-25

**Authors:** Ted Ozersky, Amanda Poste, Milla Rautio, Eva Leu

**Affiliations:** ^1^ Large Lakes Observatory and Department of Biology University of Minnesota Duluth Duluth Minnesota USA; ^2^ Norwegian Institute for Nature Research Tromsø Norway; ^3^ Département des Sciences Fondamentales, Center for Northern Studies (CEN), & Interuniversity Research Group in Limnology (GRIL) Université du Québec à Chicoutimi Québec Canada; ^4^ Akvaplan‐Niva, Fram Centre Tromsø Norway

**Keywords:** Arctic, boreal, climate change, ice, limnology, winter ecology

## Abstract

Climate warming is especially pronounced in winter and at high latitudes. Warming winters are leading to the loss of lake ice and changing snow cover on lakes. Historically, lake scientists have paid less attention to the ice cover period, leading to data and theory gaps about the role of winter conditions in lake ecosystem function and the consequences of changing winters. Here we use simple models to show that the latitudinal interaction between ice cover duration and light flux seasonality has profound and underappreciated implications for lakes. Our models focus on light and temperature, two key drivers of ecosystem processes. We show that the relative amount of light arriving in lakes during ice cover increases non‐linearly with latitude and that the light climate of high latitude lakes is much more sensitive to changing winter conditions than that of lower latitude lakes. We also demonstrate that the synchronicity between high light and warm temperatures may decrease with latitude, with implications for primary and secondary production. Our results suggest that ice loss may lead to greater relative change to productivity and biotic interactions in higher latitude lakes and also offer several testable predictions for understanding the consequences of climate‐induced changes across latitudinal gradients.

## Graphical abstract

Climate warming is particularly strong in winter and at high latitudes, driving changes in lake ice and snow cover. Using simple models, we show that the interaction between ice duration and seasonal light flux has significant and underappreciated effects on lakes. Our results suggest that high‐latitude lakes experience greater sensitivity to winter changes, with implications for productivity, biotic interactions and ecosystem function.

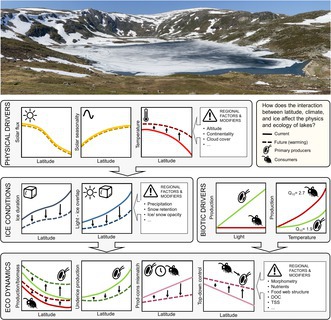

## 1 Introduction

Climate warming in the northern hemisphere is especially pronounced and rapid during winter and at high latitudes (Overland et al. 2020; IPCC (The Intergovernmental Panel on Climate Change) 2021; Saulnier‐Talbot et al. 2024). Warmer winters decrease lake ice cover duration and thickness, impact ice transparency and modify winter precipitation patterns and the accumulation of snow on lake ice (Mudryk et al. 2020; Sharma et al. 2022; Weyhenmeyer et al. 2022; Culpepper et al. 2024). Determining how these changing winter conditions affect the ecology of seasonally frozen lakes—and how lakes will respond to future winter warming—is challenging because the ice cover period is strongly underrepresented in most limnological studies.

Growing recognition of this winter knowledge gap is motivating limnologists and other environmental researchers to pay closer attention to winter and how winter conditions shape terrestrial and aquatic ecosystems and set the stage for the following seasons (Hampton et al. 2017; Berge et al. 2020; Hébert et al. 2021; Studd et al. 2021; Hampton et al. 2024). Much of this new research is necessarily ecosystem‐ and site‐specific and is only starting to produce synthetic frameworks for integrating winter into the annual ecological cycle of lakes (e.g., Cavaliere et al. 2021; Jansen et al. 2021; Yang et al. 2021). The relative scarcity of winter data, regional focus of many winter studies and gaps in theory make it difficult to detect patterns and test general hypotheses about winter ecology across broad spatial scales and to make predictions about the future of lake ecosystems.

Here we build on recent work in winter limnology to propose an explanatory framework for how winter conditions and processes integrate into the annual ecology of lakes across large latitudinal and climatic gradients. Our framework is focused on the interaction between latitudinal gradients in solar irradiance and latitudinal and regional patterns in ice and snow cover. We show that the interaction between these factors has important and not widely recognised consequences for the light and temperature regimes of seasonally ice‐covered lakes. Light and temperature are paramount physical drivers of lake ecosystem function, directly affecting primary and secondary production and the fate of organic carbon. We demonstrate that the proportion of a lake's total annual light budget that reaches the water column during the ice cover period increases non‐linearly with latitude. We also show that high latitude lakes are more sensitive to climate‐driven changes in their light climate than lower latitude lakes. Finally, we show that the synchronicity between high light levels (a key controller of primary production) and warm temperatures (which control respiration and secondary production) decreases with latitude but may become increasingly coupled with loss of ice.

Together, these results indicate that the ecological consequences of ice cover loss and other changes in winter conditions (e.g., changing ice clarity and snow conditions) will be greatest at high latitudes, with the impacts of climate change increasing from temperate to boreal to arctic lakes. Many of the elements of our proposed framework are also highly relevant for high elevation lakes, which–even at lower latitudes–typically experience long periods of ice cover.

## 2 Insolation and Ice Across Latitudes

**FIGURE 1:**
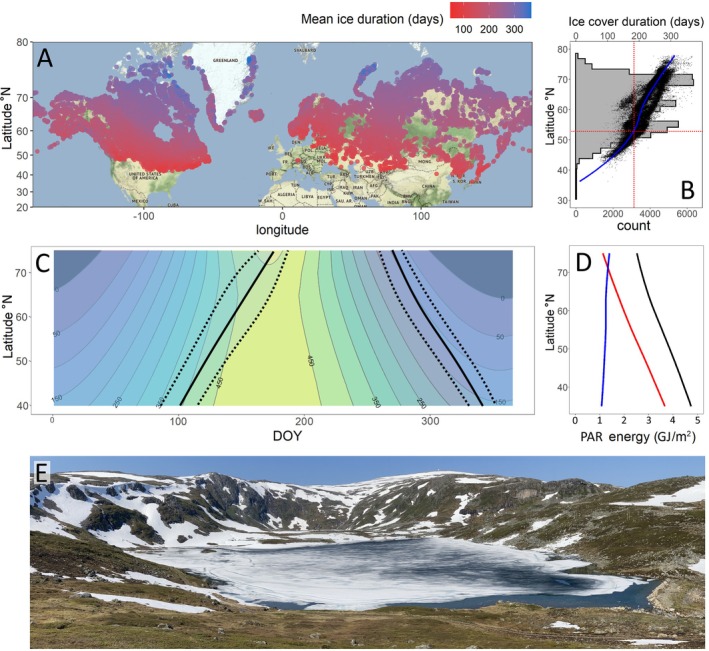
(A) ice cover duration across 67,291 lakes from Wang et al. (2022); (B) frequency distribution of seasonally‐freezing lakes (grey histogram), with ice cover duration for individual lakes (black dots) and median ice cover duration across latitudes (blue line); red dashed lines show approximate latitude where median ice cover duration = 183 days (based on results from Wang et al. 2022); (C) contour plot showing annual pattern of average daily solar flux (W/m^2^) between 30° N and 80° N with average (solid black line) and ±1SD (dotted black line) timing of ice‐on and ice‐off across the 67,291 lakes shown in panel A; (D) change in total potential (not accounting for clouds) solar PAR irradiance arriving at the Earth's surface (black line) across latitudes in relation to amount of light arriving during the typical ice cover period (blue line) and open water periods (red line) for lakes with median ice cover duration across latitudes; (E) Lake Mellomvatnet, (69.71° N, 18.82° E; 396 m A.S.L) on June 27, 2022, 1 week after the summer solstice.

From the equator to the poles, there is a 2.4‐fold decrease in total, top‐of‐atmosphere, annual solar energy flux, from ca. 13 GJ/m^2^/year at 0° to ca. 5.5 GJ/m^2^/year at 90° N/S. The seasonality of solar flux also changes with latitude, increasing in amplitude at higher latitudes. This culminates in periods of complete darkness in winter (‘polar night’) and continuous light in summer (‘midnight sun’) beyond the Arctic and Antarctic Circles (66.5°N/S). This global‐scale variation in solar energy flux drives latitudinal temperature gradients, with generally longer and colder winters at higher latitudes. More severe winters lead to earlier ice‐on, later ice‐off, thicker ice, longer ice cover duration and potentially more snow accumulation on the ice of high latitude lakes (Figure 1A,B).

Lakes are unevenly distributed across the Earth's surface. They peak in abundance between 50° N and 70° N, regions with large seasonal variation in insolation and generally cold winters (Figure 1B). It is well known that most of the world's lakes freeze for at least part of the year (e.g., Denfeld et al. 2018). What has not been as well appreciated is that, on an annual basis, most lakes that freeze are frozen for longer than they are ice free. The median ice cover duration across the world's seasonally frozen lakes is estimated to be 218 days (Wang et al. 2022) and, on average, lakes above ~55° N are frozen for more than half of the year (Figure 1B). At these high latitudes, lake ice persists late into the year and, moving poleward, increasingly overlaps with the summer period of peak light. High latitude lakes may therefore still be covered by ice during the ‘midnight sun’ period (Figure 1C). At lower latitudes, many high elevation lakes also remain frozen for large parts of calendar spring and summer.

Crucially, the latitudinal interaction between lake ice cover duration and solar flux seasonality means that, at higher latitudes, a larger fraction of annual light reaches the Earth's surface when lakes are covered by ice (Figure 1C,D). For example, at 45° N, approximately 28% of total annual solar radiation reaches the Earth's surface while lakes are ice covered. In contrast, at 75° N, approximately 56% of total annual solar radiation arrives while lakes are covered by ice (assuming median ice cover periods for both latitudes and discounting cloud cover; Figure 1C,E). This variation in the timing of incoming solar energy in relation to ice cover phenology has important consequences for lake ecology and how lake ecosystems across latitudes respond to climate change.

## 3 Ice, Snow and Light

Ice and snow reflect and attenuate light, restricting the amount of solar energy that reaches the water column of ice‐covered lakes and is available for photosynthesis and heating and mixing the water. Light transmittance by ice depends on ice clarity and albedo. Clear (‘black’) lake ice is much more transparent and less reflective than cloudy (‘white’) lake ice. A 1‐m layer of very clear, black ice (with albedo = 0.1 and a light attenuation coefficient K_d_ = 0.32 m^−1^; Belzile et al. 2001) would transmit ca. 65% of photosynthetically available radiation (PAR) reaching its surface. In contrast, 1 m of white ice with an albedo of 0.4 and K_d_ = 4 m^−1^, would transmit less than 1% of light to the water below. Snow reflects and attenuates light more efficiently than ice, and just 20 cm of snow can block 99% of PAR (Prowse and Stephenson 1986; Pernica et al. 2017), with lower transmittance for new snow with low moisture content than for wet and/or metamorphosed snow (Perovich 2007). Thus, the thickness and characteristics of ice and, even more so, snow, exert strong control on a lake's underwater light environment during winter (Cavaliere et al. 2021). In regions where black ice predominates and there is little snowfall (or limited snow retention on the ice), under‐ice light levels can be high (Prowse and Stephenson 1986; Welch et al. 1987; Imbeau et al. 2021; Bramburger et al. 2023). This is also the case both during the late ice‐cover season, when the snow has melted, as well as in response to increasingly common mid‐ and late‐winter rain‐on‐snow events (Hansen et al. 2014). On the other hand, in regions and periods characterised by thick snow and predominance of white ice, the under‐ice environment is very dark.

**FIGURE 2:**
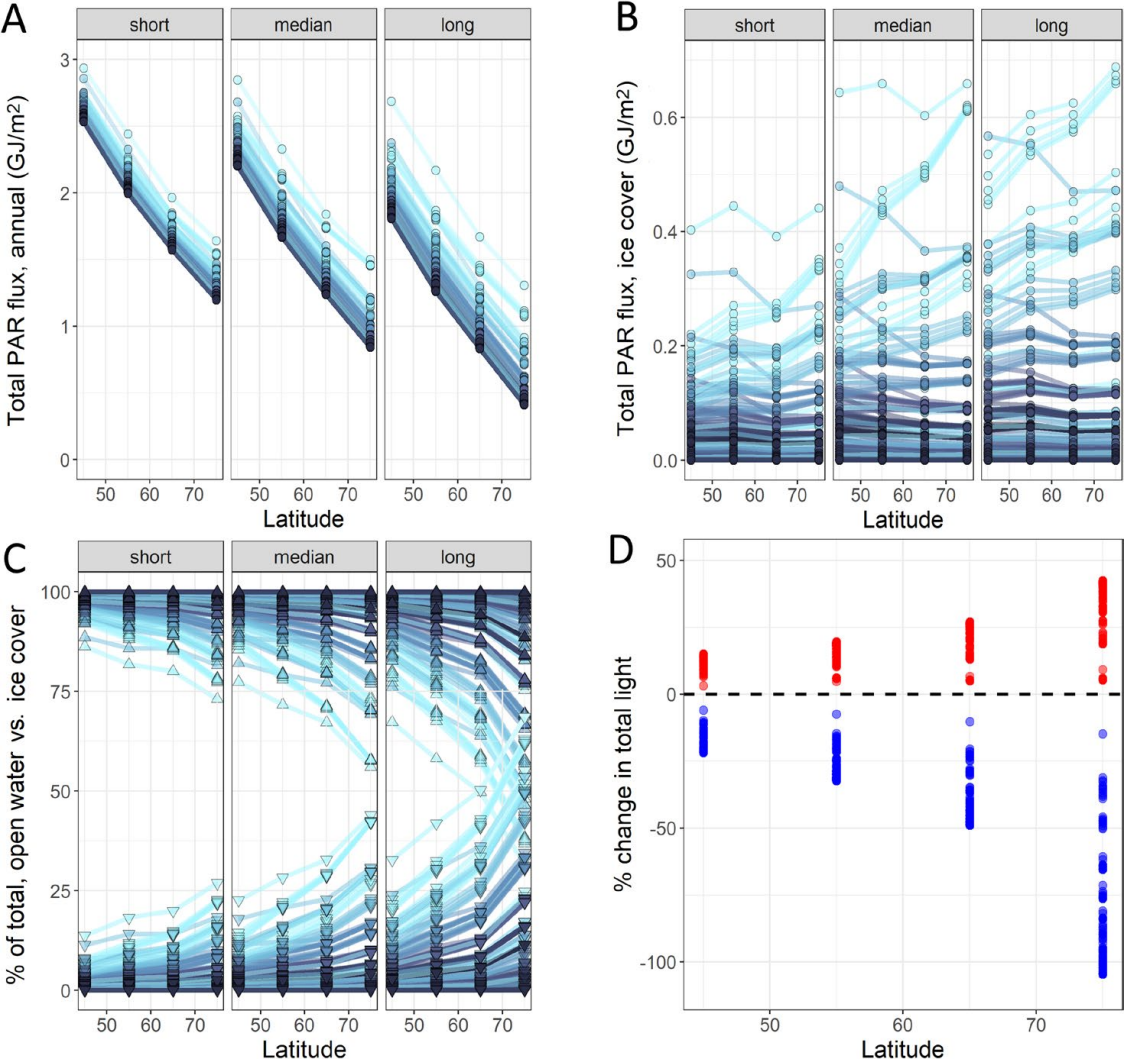
Light environment in lakes as a function of latitude and ice and snow conditions. (A) Total PAR flux reaching the water column of lakes across latitudes (45° N–75° N) under short, median and long ice cover duration scenarios for each latitude band. Points and lines represent variation due to snow cover conditions (duration and thickness) and variation in albedo and light attenuation by ice (albedo ranging from 0.1 to 0.5, K_d_ from 0.32 to 5). Line colour denotes transmission conditions through ice, with lighter blue indicating high transmission (low albedo and K_d_) and darker blue indicating low transmission (high albedo and K_d_). (B) Same as in A, but showing total flux reaching the water column during the ice cover period. (C) The relative portion of total annual light flux reaching the water column during the open water period (lines and upward‐pointing triangles along top portion of panel) versus during the ice cover period (lines and downward‐pointing triangles along bottom portion of panel); interpretation of shading as in panels A and B. (D) Percent change in the total annual light level arriving into a lake among median (black dashed line), short (red points) and long (blue points) ice cover duration scenarios across latitudes.

At higher latitudes, increasing solar flux seasonality and a greater overlap between the ice‐cover period and peak solar irradiance make ice and snow conditions increasingly important for lake light regimes and total annual light budgets. We illustrate these effects using simple models where we combine realistic ice and snow cover phenologies, albedos, light attenuation coefficients and solar flux patterns to examine how the light environment of lakes changes across latitude and gradients in winter climate conditions (Figure 2A,B). We modelled the year‐round light environment (i.e., the light flux reaching the top of the water column) of seasonally frozen lakes at 45° N, 55° N, 65° N and 75° N. We examined median, short (median minus 1 month) and long (median plus 1 month) ice cover durations, based on the northern hemisphere lake ice phenology data from Wang et al. (2022). Short and long ice cover periods were included to explore how variation in ice cover duration, whether driven by anthropogenic factors (climate change‐driven ice loss) or regional climate (elevation, continentality) affects light flux. Seasonal ice and snow thickness and phenology were simulated based on literature data, published model results and personal observations (e.g., Greenbank 1945; Schindler et al. 1974; Welch et al. 1987; Dibike et al. 2012; Leppäranta 2015; Grosbois et al. 2017; Yang et al. 2020; Xie et al. 2023; Ghane and Boegman 2023; Kers et al. 2024). We used a range of empirically determined albedo and light attenuation coefficient values for ice and snow to model light transmittance. This approach enabled us to evaluate total lake light budgets as well as the amount of light reaching the top of the water column during the open water and ice cover periods across the modelled scenarios. Additional details on modelling and example values and ranges are presented in the Supporting Information section.

Our models reveal several important and robust patterns. First, and not unexpectedly, the total annual light flux reaching the water column of lakes across the full year decreases with increasing latitude and ice cover duration, as well as with more opaque ice conditions and thicker snow cover (Figure 2A). Second, the total light flux reaching the water column during the ice cover period tends to decrease with latitude, but only slightly (Figure 2B). Interestingly, under conditions of minimal snow cover and high ice transparency, total under‐ice light flux during the ice cover period can actually increase with latitude. This increase is due to a greater overlap between the ice cover season and periods of higher solar flux in late spring and early summer as ice cover duration increases. For the same reason, total light flux during ice cover also increases with longer ice cover duration within each latitude band (Figure 2B). The total solar flux reaching the water column during the ice cover period is very strongly influenced by ice transparency and snow cover conditions, with the impact of snow and ice conditions increasing with ice cover duration. This results in striking variability in under‐ice light levels, even among lakes at the same latitude (Figure 2B), and suggests that climate‐driven changes in snow and ice properties during winter could have major consequences for lake light regimes.

Third, the relative amount of total annual light reaching the water column during the ice cover season increases with latitude and ice cover duration (Figure 2C). In other words, lakes at high latitudes and with longer ice cover duration for a particular latitude band can receive more of their total annual light budget when they are covered by ice. In our models, lakes at 75° N received up to 69.5% of their total annual light budget during ice cover (range = 0%–69.5% and mean = 12.5% across all simulation scenarios). In contrast, lakes at 45° N received no more than 28% of their total annual light budget during ice cover (range of 0%–28.0% and mean of 4.2%). Across latitudes, snow depth and duration played a key role in determining this quantity. Given the strong seasonality of cloud cover in the Arctic (high cloud cover in summer and early autumn and low cloud cover in late spring; Liu and Schweiger 2017), our approach may even underestimate the amount of light reaching high latitude lakes during their ice‐cover period.

Finally, the effects of natural and climate change‐induced variation in ice cover duration are also magnified with latitude (Figure 2D). Because a larger fraction of the annual light budget arrives during the ice cover period at high latitudes, a 1‐month difference in ice cover duration has a larger effect on a lake's under‐ice and total annual light budget (as well as the ratio between light arriving into the lake during the ice cover and open water periods) at higher latitudes. This variation in the effects of ice cover loss means that an equal duration of ice cover loss will have a different impact on lakes across latitudes or lakes that experience different winter severity. In particular, high latitude lakes and lakes that have especially long ice cover periods (e.g., because of a strongly continental climate, ice cap climate or high elevation) will experience much larger increases in their annual light budgets than lakes with shorter ice cover duration. For example, a loss of 1 month of ice cover (15‐day earlier ice‐off in spring and 15‐day later ice‐on in fall) for a lake with median ice cover duration at 45° N (142 days) will lead to a 3.1%–15% (mean = 13.0%) increase in total annual light flux; an equivalent loss of ice cover for a lake that has an extra month of ice cover duration at 45° N will lead to a 5.9%–21.8% (mean = 19.4%) increase in total annual light. In contrast, similar losses of ice for a lake at 75° N would result in an increase of 5.4%–42.4% (mean = 35.3%) in annual light flux for a lake with a median (270 days) ice cover duration and of 14.8%–105% (mean = 80.3%) for one that has an extra month of ice cover. The degree of increase in total light will be strongly related to snow depth and ice clarity, with the greatest increases for lakes that experience deep snow cover and predominance of white ice.

## 4 Light and Temperature

**FIGURE 3:**
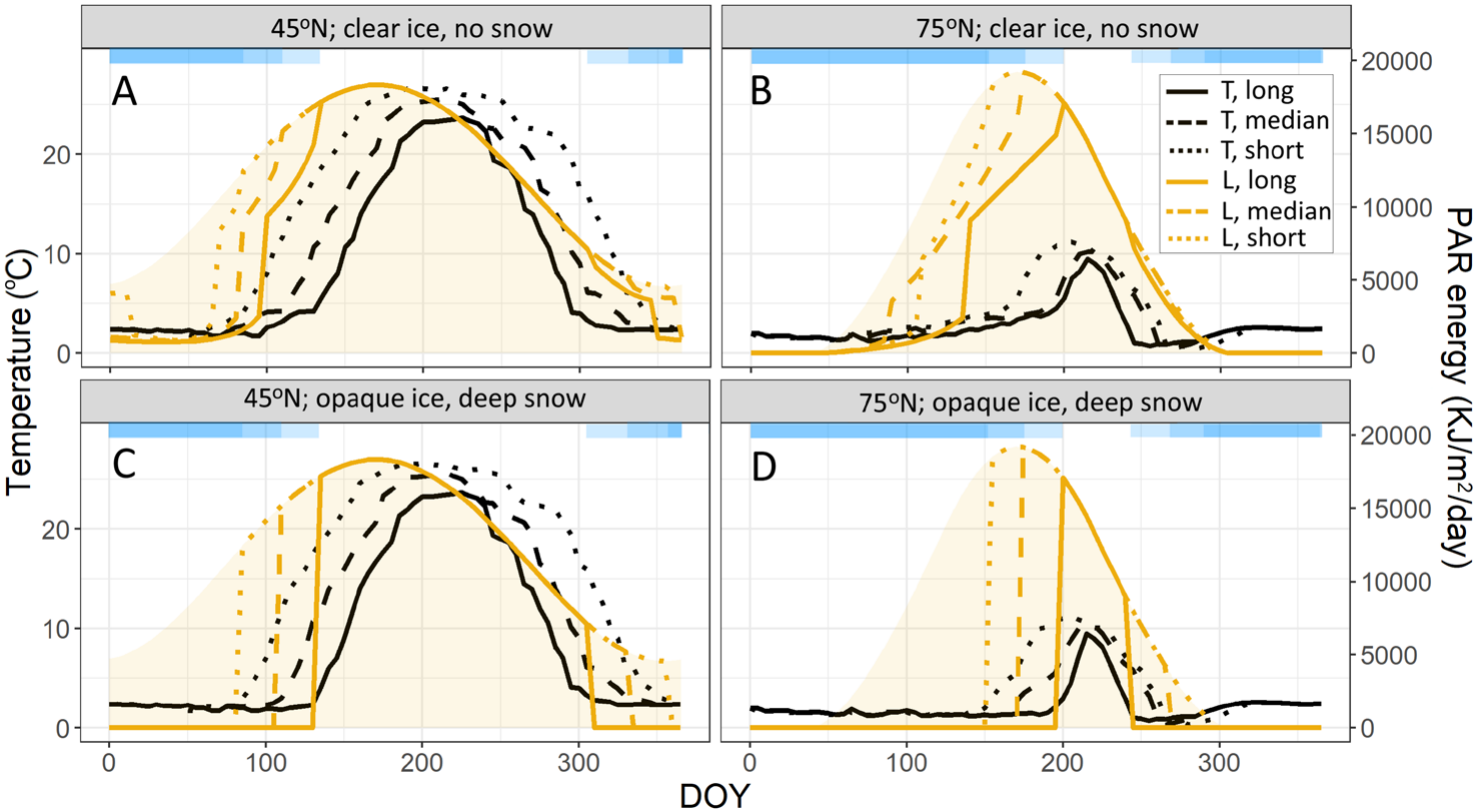
Seasonality and predicted effects of variation in ice cover duration and ice/snow conditions on light and temperature in hypothetical lakes at 45° N and 75° N. (A) Seasonal PAR flux (light yellow background), under‐ice light conditions (yellow lines), and surface temperature (black lines) at 45° N with clear ice (K_d_ = 0.32), snow cover for 60% of the ice period, and maximum snow depth of 5 cm. (B) Same as A, but at 75° N. (C) Same as A, but with opaque ice (K_d_ = 5), snow cover for 100% of the ice period and maximum snow depth of 80 cm. (D) Same as C, but at 75° N. Dotted, dashed, and solid lines represent short, median and long ice cover durations, respectively. Light blue blocks show ice cover duration for the three ice cover duration scenarios.

Latitudinal patterns of solar flux and ice cover seasonality interact with lake temperature dynamics. To illustrate these interactions across latitudes, we compare two hypothetical lakes at 45° N and 75° N that differ in ice duration and snow cover conditions (Figure 3). In‐lake light levels are based on our modelled seasonal light flux across varying ice durations (short, median and long) and two contrasting ice and snow scenarios: one with highly transparent ice, thin snow cover and short snow retention (Figure 3A,C); the other with opaque ice and deep, persistent snow (Figure 3B,D). To represent annual temperature dynamics, we use published full‐year data from a temperate lake and a high Arctic lake (Tasnim et al. 2021; Grosbois et al. 2022), modifying them for short and long ice cover scenarios based on the timing of ice‐off and ice‐on and adjusting peak temperatures to reflect changes in open water duration. Though not representative of all lakes at their latitudes, these examples illustrate key contrasts in light and thermal regimes and how they are affected by variability in ice and snow conditions.

While radiative heating of under‐ice water does contribute to lake heat budgets and drives under‐ice mixing, the bulk of the water column under ice rarely exceeds 4°C, and most heating happens after ice‐off (Vincent et al. 2008; MacIntyre and Hamilton 2024). At temperate latitudes, ice‐off generally occurs relatively early in spring, when light levels are still increasing toward their annual maximum. At these latitudes, water temperature in most lakes increases rapidly after ice‐off, stratification often sets in quickly and the epilimnion continues to gain heat through the summer, reaching high temperatures by July (Figure 3). For example, in central Minnesota (USA, 45° N), ice‐off typically occurs in late March, stratification sets up within 2–3 weeks, and peak summer temperatures reach 25°C or more (e.g., Tasnim et al. 2021).

Water temperatures and the duration of summer stratification decrease with latitude (Woolway et al. 2021; Korver et al. 2024), and the later persistence of ice means ice cover overlaps more closely with the annual peak of solar flux than at lower latitudes (Figure 3). Arctic and subarctic lakes can exhibit diverse thermal and stratification regimes (Vincent et al. 2008; Cortés and MacIntyre 2020; MacIntyre and Hamilton 2024) and may reach peak temperatures of 15°C or more (e.g., Laurion et al. 2010). However, in many lakes above 70° N, peak summer temperatures rarely exceed 10°C (e.g., Ayala‐Borda et al. 2024). High latitude lakes that stratify do so later and are stratified for shorter periods than boreal and temperate lakes, and many Arctic lakes are cold monomictic or polymictic, with only transient stratification during their brief ice‐free periods (Schindler et al. 1974; Welch et al. 1987; Sorvari et al. 2000; MacIntyre et al. 2009; Saros et al. 2016; MacIntyre and Hamilton 2024).

**FIGURE 4:**
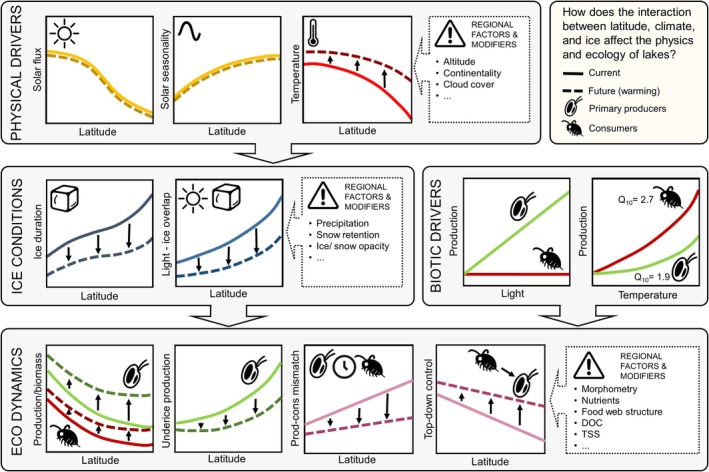
Conceptual summary of proposed physical‐ecological linkages across latitudes in seasonally freezing lakes, shown in four panels (top to bottom, left to right). PHYSICAL DRIVERS: Latitudinal patterns in total solar flux, seasonality of solar flux, air/water temperatures and potential regional modifiers. ICE CONDITIONS: Latitudinal patterns in duration of ice cover and in the proportion of annual solar radiation arriving during ice cover, with potential regional modifiers. BIOTIC DRIVERS: dependence of community‐level primary and secondary production on light and temperature. ECO DYNAMICS: Latitudinal patterns in primary and secondary production and biomass, proportion of total primary production occurring under ice, the degree of mismatch between peak primary and secondary (consumer) production, the strength of top‐down control on primary producers and potential regional modifiers. Solid lines indicate current conditions and dashed lines show potential changes with climate warming; black arrows represent climate‐driven directional shifts.

These differences in the timing of water heating and solar flux lead to latitudinal variation in the synchronicity between light and warm temperatures. At lower latitudes, peak light and warm temperatures are closely coupled. In central Minnesota, the period of peak irradiance occurs when lakes are stratified and the surface temperature is > 20°C (e.g., Tasnim et al. 2021; Figure 3A,C). At high latitudes, the timing of peak light and temperature is more decoupled (Figure 3B,D). Irradiance peaks during late ice cover and shortly after ice‐off when most lakes are mixed and the water column is still cold. By the time lakes at 70° N reach their modest maximum surface temperatures in August, light levels have already decreased markedly compared to their June peak. Thus, the portion of the annual light budget that arrives when water temperatures are low is greater at high latitudes. The degree of overlap between illumination and warmth is also more sensitive to variation in snow depth and ice opacity in high latitude lakes, as deep snow and white ice would delay a larger portion of the annual incoming light until ice‐off. Because light and temperature are primary physical controllers of biological productivity, their latitudinal variation and synchronicity will have important consequences for lake ecosystems (Figure 4).

## 5 Consequences for Biology

Light is the main physical driver of aquatic primary production, with temperature becoming increasingly important under light‐saturated conditions (Tilzer et al. 1986; Fahnenstiel and Scavia 1987; Sterner 2010; Edwards et al. 2016; Sherman et al. 2016). Appreciable rates of benthic and pelagic primary production—and even large phytoplankton blooms—have been observed from many ice‐covered lakes and seas whenever sufficient light reaches the water (Welch and Kalff 1974; Straškrábová et al. 2005; Salmi and Salonen 2016; Ardyna et al. 2020; Bramburger et al. 2023). In lakes, underice phytoplankton blooms are often composed of large, low‐light adapted diatoms that rely on light‐driven convective mixing to remain in suspension, or motile taxa that can overcome mixing to maintain their position close to the bottom of the ice (Kelley 1997; Jewson et al. 2009; Kiili et al. 2009; Yang et al. 2020; Jansen et al. 2021). In contrast to primary producers, invertebrate metabolism, activity, growth and reproduction strongly increase with temperature (e.g., Brown et al. 2004). Temperature predicts aquatic invertebrate production rates (Plante and Downing 1989; Huntley and Lopez 1992; Hansen et al. 1997; Shuter and Ing 1997) as well as abundances within (Watson and Wilson 1978; Herzig et al. 1980; Carter and Schindler 2012; Cremona et al. 2020; Shchapov and Ozersky 2023) and across systems (Patalas 1975; Kitaev 1984; Loria et al. 2020; Kelly and Jones 2023). These differences are reflected in lower temperature sensitivity coefficients (Q_10_ values) for phytoplankton (Q_10_≈1.5–1.9; Sherman et al. 2016) compared to aquatic invertebrates (Q_10_≈2–3 or more; Huntley and Lopez 1992; Tumbiolo and Downing 1994; Hansen et al. 1997). Thus, seasonal variation in irradiance and temperature across latitudes should result in predictable latitudinal patterns in the timing and magnitude of primary and secondary production and their interaction (Figure 4).

Because of lower total solar flux, its greater seasonality and longer ice cover, primary production in high latitude lakes is lower and more seasonally variable compared to lower latitudes (Brylinsky and Mann 1973; Boulion 1994; Lewis Jr. 2011). Longer ice cover and its greater overlap with peak solar irradiance also mean that the proportion of total annual primary production that occurs under the ice and shortly after ice‐off, when water temperatures are low, may increase with latitude (Figure 4). The overlap between peak irradiance and ice cover at high latitudes also suggests that climate change‐driven loss of late‐season ice may result in larger potential increases in total annual primary production in higher latitude lakes (Figure 4). For the same reason, variation in snow cover conditions and ice clarity should also have a larger impact on primary productivity at high latitudes. At lower latitudes, ice loss should have a smaller impact on pelagic and benthic primary production because the resulting increase in incoming light –in both absolute and relative terms– is less pronounced than at higher latitudes (Figure 2D).

Integrated annual consumer production in lakes is generally assumed to decrease with increasing latitude because of decreasing average annual temperatures and primary productivity (e.g., Plante and Downing 1989; de Senerpont Domis et al. 2013). Although few large‐scale syntheses exist, Kitaev (1984) documented increasing zooplankton and benthos abundance along a latitudinal gradient from tundra to boreal to temperate lakes. Animals in high latitude lakes experience long periods of low temperatures, darkness and low plant food availability during late fall and most of winter. At 75° N, the open water period of most lakes is brief and peak water temperatures are low. Thus, high latitude lakes should generally produce less zooplankton and benthos biomass across the year, with lower peak summer abundances, compared to lower latitude lakes (Rigler et al. 1974; Patalas 1975; Welch 1976; Kitaev 1984; de Senerpont Domis et al. 2013). The long period of unfavourable winter conditions in high latitude lakes should also result in greater overwinter mortality of zooplankton (Hébert et al. 2021) and higher reliance on diapause rather than active overwinter survival, resulting in lower zooplankton abundance through the winter and into the early period of phytoplankton growth as light levels increase (Figure 4).

The partly independent effects of light and temperature on primary and secondary production (e.g., Pomeroy and Deibel 1986; Gronchi et al. 2023) should result in an increasing mismatch between the timing of peak primary and secondary production with latitude. At lower latitudes, water temperatures tend to be relatively high during the period of peak solar flux in late June (Figure 3). At high latitudes, there generally is a greater lag between high light and maximum water temperatures, with peak solar flux occurring when water temperatures are still around 4°C or lower (e.g., if lakes are still ice‐covered). This latitudinal mismatch between high temperature and light should have implications for producer–consumer interactions. Under current conditions, planktonic and benthic grazers should exert less top‐down pressure on spring and summer phytoplankton and periphyton in high latitude lakes, where under‐ice and early spring plant growth occurs at low temperatures that are less favourable to consumer activity (Figure 4). However, with ongoing climate change and ice loss, the synchronicity of light and temperature at high latitudes will increase, resulting in a higher potential for top‐down control of spring primary production (Pomeroy and Deibel 1986; O'Connor 2009; Gutow et al. 2016). In regions where climate change is leading to increased snow cover, deep snow (or opaque ice) can delay substantial primary production until later in spring, also potentially increasing the synchronicity between primary and secondary production relative to lakes with little snow and clear ice (Figure 3).

## 6 Caveats and Conclusions

To focus on the role of ice cover in shaping lake ecosystems, we examined how light regimes are shaped by ice and snow conditions across large latitudinal gradients. We combined our estimates of light phenology with temperature data to illustrate how these abiotic factors may shape the productivity and abundance of primary producers and consumers. Our analysis emphasises large‐scale latitudinal patterns, but many physical, chemical and biological factors operate at regional and local scales and play important roles in structuring lake ecosystems. These factors will interact with the broad latitudinal patterns we describe to shape conditions in individual lakes (Figure 4).

Our estimates of underwater light availability carry uncertainties related to seasonal and regional variation in cloud cover, snow phenology and characteristics, water colour and turbidity. While latitude is a key driver of lake temperatures, it is also affected by local conditions such as lake size, depth, elevation, and regional climate. Other factors—such as nutrient supply, vertical mixing and community structure—interact with snow and ice conditions to influence primary producers, invertebrate consumers and their interactions (Figure 4). For example, mixing regimes strongly affect light availability for phytoplankton, particularly in deep or highly stained lakes, and—like ice cover—the timing, depth and duration of stratification are sensitive to climate change (Sommer et al. 2012; Pernica et al. 2017; Bouffard et al. 2019; Ghane and Boegman 2025). Nutrient concentrations generally decrease with latitude due to less intense land use and lower human population density, contributing to large‐scale variation in nutrient limitation and productivity (Boulion 1994; Lewis Jr. 2011; Abell et al. 2012). Other organisms, including mixotrophs and members of the microbial loop, play important but still poorly quantified roles in lake ecosystems, particularly under ice where their contributions to energy flow and nutrient cycling may be high. Fish are absent from many shallow high latitude lakes due to complete freezing of the water column, and absence of fish predation can lead to high abundances of zooplankton and benthic invertebrates despite low temperatures and nutrient concentrations (Christoffersen et al. 2008; Rautio et al. 2008).

We make three key claims: (1) light conditions in high latitude lakes are more sensitive to ongoing and predicted changes in snow and ice conditions than in lakes at lower latitudes; (2) the relative contribution of under‐ice and cold water period primary production increases with latitude; and (3) high‐latitude lakes will experience greater relative changes in productivity and biotic interactions, including decreased synchronicity between primary and secondary production, in response to climate‐driven variations in winter conditions. These patterns will be exacerbated by the more rapid warming and loss of ice that high latitude lakes are experiencing compared to lakes at lower latitudes (Saulnier‐Talbot et al. 2024). While our models make many generalisations and simplifying assumptions, they are based on well‐established principles and suggest novel and testable predictions about the full year seasonality of lakes across broad latitude gradients, including the role of winter and ice cover in shaping lake ecosystems. There is considerable potential to test, refine and extend these predictions with better models and coordinated studies of the full‐year seasonality of lakes across gradients of latitude and winter severity.

## Author Contributions

Ted Ozersky and Amanda Poste conceived the manuscript and Ted Ozersky, Amanda Poste, Milla Rautio, and Eva Leu developed the ideas. Ted Ozersky led writing of the text, with input from Amanda Poste, Milla Rautio, and Eva Leu. Ted Ozersky performed modelling and data visualization with input from Amanda Poste, Milla Rautio, and Eva Leu.

## Conflicts of Interest

The authors declare no conflicts of interest.

## Peer Review

The peer review history for this article is available at https://www.webofscience.com/api/gateway/wos/peer‐review/10.1111/ele.70200.

## Supporting information


**Data S1:** ele70200‐sup‐0001‐Supinfo.docx.

## Data Availability

The data and R code used to support the findings of this study are archived on Dryad and available at: https://doi.org/10.5061/dryad.98sf7m0wc
