# Supplementary material for: Impacts of Changing Winters on Lake Ecosystems Will Increase With Latitude
Source: Ecol Lett. 2025 Aug 25;28(8):e70200. doi: 10.1111/ele.70200 (PMC12376094; doi:10.1111/ele.70200)
Supplement: Supplementary file 1 — Data S1: ele70200‐sup‐0001‐Supinfo.docx. [file ELE-28-0-s001.docx]

**Supplementary information section**

***Model details:***

Ice and snow cover scenarios: to assess the effect of snow and ice cover on the light climate of lakes across latitudes, we constructed ice and snow cover scenarios for lakes in 10° latitude intervals (45, 55, 65, 75 °N). Ice cover duration, ice-on, and ice-off dates were determined based on values in the Global Annual Lake Ice Phenological Dataset 1861-2099 (Wang et al. 2022). The dataset contains satellite-derived ice phenology data for the years 2001-2020 for 74,245 global lakes. We trimmed the dataset by excluding lakes that did not freeze every year during the 20 years of the observation period, lakes that were south of 30 °N, and lakes at elevation of more than 1,000 m above sea level (ASL), leaving us with a set of 67,291 observations. We averaged ice-on, ice-off and ice-cover duration data for the available 20 years for each of the lakes. Few low-elevation lakes below ~40 °N regularly froze, so further analyses were restricted to the 45-75 °N latitude interval.

Ice thickness evolution for each latitude (e.g., 45 °N) was modelled based on the median ice-on and ice-off dates for lakes in the corresponding 10 ° latitude band (e.g., 40-50 °N), a wide range of literature data on empirical and modelled ice evolution in diverse lakes (Greenbank 1945; Schindler et al. 1974; Welch et al. 1987; Dibike et al. 2012; Leppäranta 2015; Grosbois et al. 2017; Yang et al. 2020; Clark et al. 2022; Xie et al. 2023; Ghane & Boegman 2023; Shchapov & Ozersky 2023), and the author’s personal observations. Ice growth in fall/winter and its destruction in the spring were modelled as a 4th-order polynomials (Fig. S1), with maximum thickness increasing from 0.7 to 1.5 m from 45 to 75 °N. In addition to these ‘median’ scenarios, we created ice cover models for a hypothetical ‘long’ ice cover season for each latitude (e.g., for a higher elevation locations or locations with a cold continental climate), and ‘short’ ice cover season scenarios, as might be expected under continuing climate change or a relatively warm maritime climate. The ‘long’ and ‘short’ scenarios have 1-month longer and 1-month shorter ice cover scenarios compared to the median scenarios, with lakes in the ‘long’ scenarios having a 15-day earlier ice-on and 15-day later ice-of than the ‘median’ lakes. Similarly, the ‘short’ scenario lakes have 15-day later ice-on and 15-day earlier ice-off than ‘median’ lakes. These ‘long’ and ‘short’ ice cover scenarios allowed us to assess how lakes with different ice cover duration and at different latitudes compare and respond to climate-driven ice loss. The maximum ice thickness for each latitude bands was increased or decreased by 20 cm for the ‘long’ and ‘short’ scenarios, respectively to represent the decreased ice thickness that typically corresponds to shorter ice cover duration.

Snowfall amounts and the accumulation of snow on lakes are highly variable on global and regional scales (Kunkel et al. 2016; Pulliainen et al. 2020; Dou et al. 2021). We modelled several snow cover scenarios for all of the above-described ice cover scenarios. Three basic scenarios were examined: 60%, 80%, and 100% scenarios corresponded to snow cover over the ice for 60%, 80%, and 100% of the ice cover season, centered on the middle of the ice cover period. Thus, for 60% scenarios, the first and last 20% of the ice cover period were snow free, and so on. The near absence of snow over the ice at the start and end of the ice cover season is typical of many locations. For each snow-cover duration scenario we also varied maximum snow thickness in 5 cm increments, from 0 cm to 80 cm. Snow accumulation and melting was modelled using 3rd order polynomial equations (Fig. S1). We also included an extreme case scenario, when a thick layer of snow was deposited on the ice for the entire duration of the ice cover period. Thus, our snow cover scenarios capture diverse conditions between the two extremes of completely snow-free ice (e.g., lakes in cold desert regions) and ice that is covered by a thick blanket of snow for the entire period of ice cover (e.g., high elevation lakes).

Underice light climate: We used our snow and ice cover scenarios to model the potential light climate under ice and snow cover for lakes across latitudes and with varying ice cover and snow cover regimes. We obtained daily cloud-free solar flux data (as W/m^2^) for 45, 55, 65, and 75 °N from NASA’s ModelE AR5 Insolation at Specified Location website (NASA 2023). Daily average solar flux data as W/m^2^ were converted solar flux energy values in MJ/m^2^/day and to photosynthetically active radiation (PAR) energy assuming that 0.43 of total solar energy is in the visible range and thus available for photosynthesis. These PAR energy values were combined with daily modelled ice and snow thickness scenarios and realistic ice and snow albedos and light attenuation coefficients (k_d_) to determine the amount of solar energy penetrating the snow and ice cover and entering the unfrozen water (Fig. S2). Light penetration through ice and snow was modelled using a two-compartment model (e.g., Prowse & Stephenson 1986; Equation 1), with snow and ice having separate albedo and k_d_ values. When snow was present on the ice, we modelled underice light by accounting for reflection of incoming light from the surface of the snow, attenuation by the snow layer, and then attenuation by the ice layer (assuming no additional reflection from the surface of the ice layer). When the ice was snow-free, we modelled light penetration based on reflection from the surface of the ice and attenuation by the ice layer.

Equation 1: $I=Io\cdot\left( 1-\alpha_{\mathrm{snow}} \right)\cdot e^{{-k}_{snow}}\cdot^{z_{snow}}\cdot\left( 1-\alpha_{\mathrm{ice}} \right) \cdot e^{{-k}_{ice}}\cdot^{z_{ice}}$

Where:

- I = under-ice PAR flux
- I_0_​ = incoming flux at the snow surface
- α_snow_ = albedo of snow
- α_ice_ ​ = albedo of ice
- k_snow_ = attenuation coefficient for snow (m⁻¹)
- k_ice_= attenuation coefficient for ice (m⁻¹)
- z_snow_= snow depth (in meters)
- z_ice_​ = ice thickness (in meters)

Snow albedo and light attenuation coefficients were set at 0.7 and 15, respectively, representing an intermediate albedo and attenuation coefficient value reported in the literature for snow. We varied ice albedo and attenuation to include variation in ice quality in our estimates of underice light levels. Ice albedo and k_d_ were varied from albedo (a)=0.1 and k_d_=0.32 (black, very clear clear ice with very low reflectance) to albedo=0.5 and k_d_ = 5 (white ice with high reflectance). The following combination of values were used: a=0.1, k_d_=0.32; a=0.1, k_d_=1; a=0.2, k_d_=2; a=0.3, k_d_=3; a=0.4, k_d_=4; a=0.5, k_d_=5. Ice and snow albedo values and attenuation coefficients were based on published literature values (Bolsenga 1969; Mellor 1977; Adams 1978; Prowse & Stephenson 1986; Belzile et al. 2001; Leppäranta et al. 2012; Warren 2019). Modeled under-ice levels based on these albedo and attenuation values were in good agreement with diverse empirical observations (e.g., Schindler et al., 1974; Welch et al. 1987; Belzile et al., 2001; Lei et al., 2011; Bramburger et al., 2023; Shchapov and Ozersky 2023). For example, 0.5 m of snow-free ice in our models transmitted between ~4 and 55% of the light reaching its surface, depending on the K_d_ and albedo of ice (albedo range=0.1–0.4, K_d_ range=1–5). Just 5 cm of snow over white ice (albedo=0.4, K_d_=4), reduced under-ice light levels to <2% of surface light. Forty cm of snow over 0.5 m of ice, permitted less than 0.1% of incident light to reach the unfrozen water. The combination of solar flux data with modelled ice and snow phenology allowed us to determine how much solar radiation arrived into lakes during the open-water and the ice-cover period across diverse ice and snow cover scenarios and to assess how variation in snow cover and ice duration affects the light budget of lakes across latitudes.

***Methods references:***

Adams, W. A. (1978). Effects of ice cover on the solar radiation regime in Canadian lakes: With 8 figures and 1 table in the text. Internationale Vereinigung für theoretische und angewandte Limnologie: Verhandlungen, 20(1), 141-149.

Belzile, C., Vincent, W. F., Gibson, J. A., & Hove, P. V. (2001). Bio-optical characteristics of the snow, ice, and water column of a perennially ice-covered lake in the High Arctic. Canadian Journal of Fisheries and Aquatic Sciences, 58(12), 2405–2418.

Bolsenga, S. J. (1969). Total albedo of Great Lakes ice. Water Resources Research, 5(5), 1132-1133.

Clark, J. A., Jafarov, E. E., Tape, K. D., Jones, B. M., & Stepanenko, V. (2022). Thermal modeling of three lakes within the continuous permafrost zone in Alaska using the LAKE 2.0 model. Geoscientific Model Development, 15(19), 7421-7448.

Dibike, Y., Prowse, T., Bonsal, B., Rham, L. D., & Saloranta, T. (2012). Simulation of North American lake‐ice cover characteristics under contemporary and future climate conditions. International Journal of Climatology, 32(5), 695–709.

Dou, T., Xiao, C., Liu, J., Wang, Q., Pan, S., Su, J., ... & Eicken, H. (2021). Trends and spatial variation in rain-on-snow events over the Arctic Ocean during the early melt season. The Cryosphere, 15(2), 883–895.

Ghane, A., & Boegman, L. (2023). The dissolved oxygen budget of a small Canadian Shield lake during winter. Limnology and Oceanography, 68(1), 265–283.

Greenbank, J. (1945). Limnological conditions in ice-covered lakes, especially as related to winter-kill of fish. Ecological Monographs, 15(4), 343–392.

Grosbois, G., Mariash, H., Schneider, T., & Rautio, M. (2017). Under-ice availability of phytoplankton lipids is key to freshwater zooplankton winter survival. Scientific Reports, 7(1), 11543.

Kunkel, K. E., Robinson, D. A., Champion, S., Yin, X., Estilow, T., & Frankson, R. M. (2016). Trends and extremes in Northern Hemisphere snow characteristics. Current Climate Change Reports, 2, 65–73.

Leppäranta, M. (2015). Freezing of lakes and the evolution of their ice cover. Springer.

Leppäranta, M., Heini, A., Jaatinen, E., & Arvola, L. (2012). The influence of ice season on the physical and ecological conditions in Lake Vanajanselkä, southern Finland. Water Quality Research Journal of Canada, 47(3–4), 287–299.

Mellor, M. (1977). Engineering properties of snow. Journal of Glaciology, 19(81), 15-66.

NASA. (2023). ModelE AR5 Insolation at Specified Location. Available at: https://data.giss.nasa.gov/modelE/ar5plots/srlocat.html

Prowse, T. D., & Stephenson, R. L. (1986). The relationship between winter lake cover, radiation receipts and the oxygen deficit in temperate lakes. Atmosphere-Ocean, 24(4), 386–403.

Pulliainen, J., Luojus, K., Derksen, C., Mudryk, L., Lemmetyinen, J., Salminen, M., ... & Norberg, J. (2020). Patterns and trends of Northern Hemisphere snow mass from 1980 to 2018. Nature, 581(7808), 294-298.

Schindler, D. W., Welch, H. E., Kalff, J., Brunskill, G. J., & Kritsch, N. (1974). Physical and chemical limnology of Char Lake, Cornwallis Island (75°N lat.). Journal of the Fisheries Board of Canada, 31(5), 585–607.

Shchapov, K., & Ozersky, T. (2023). Opening the black box of winter: Full‐year dynamics of crustacean zooplankton along a nearshore depth gradient in a large lake. Limnology and Oceanography, 68, 1438–1451.

Wang, X., Feng, L., Qi, W., Cai, X., Zheng, Y., Gibson, L., ... & Bryan, B. A. (2022). Continuous loss of global lake ice across two centuries revealed by satellite observations and numerical modeling. Geophysical Research Letters, 49(12), e2022GL099022.

Warren, S. G. (2019). Optical properties of ice and snow. Philosophical Transactions of the Royal Society A, 377: 20180161.

Welch, H. E., Legault, J. A., & Bergmann, M. A. (1987). Effects of snow and ice on the annual cycles of heat and light in Saqvaqjuac Lakes. Canadian Journal of Fisheries and Aquatic Sciences, 44(8), 1451–1461.

Xie, F., Lu, P., Leppäranta, M., Cheng, B., Li, Z., Zhang, Y., ... & Zhou, J. (2023). Heat budget of lake ice during a complete seasonal cycle in lake Hanzhang, northeast China. Journal of Hydrology, 620, 129461.

Yang, B., Wells, M. G., McMeans, B. C., Dugan, H. A., Rusak, J. A., Weyhenmeyer, G. A., ... & Young, J. D. (2021). A new thermal categorization of ice‐covered lakes. Geophysical Research Letters, 48(3), e2020GL091374.

***Supplementary figures:***


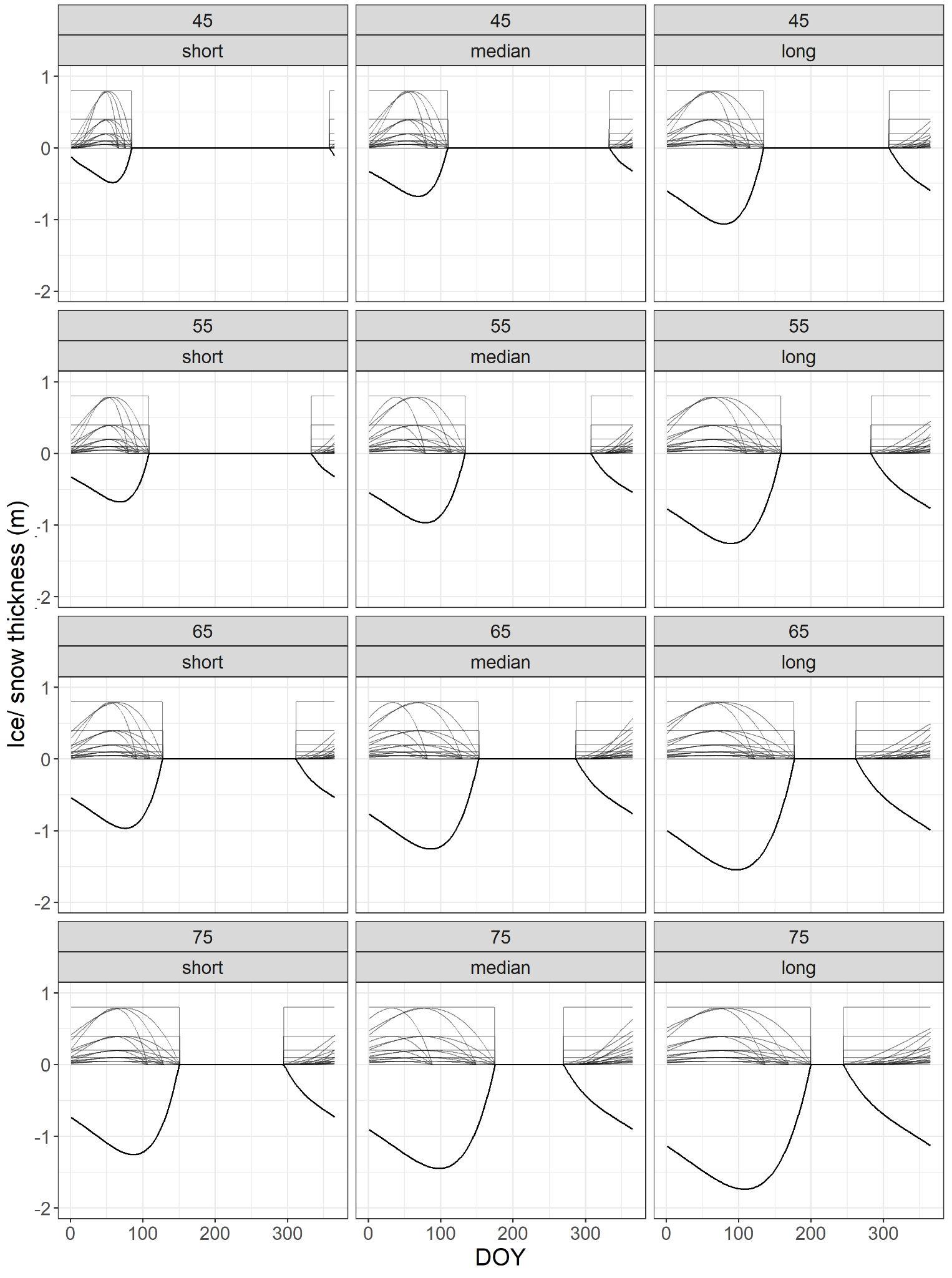
**Fig S1**: Modelled ice (negative values) and snow (positive values) thickness scenarios across latitudes and ice cover duration ranges. These ice and snow scenarios, along with realistic snow and ice light attenuation values, were used to model under-ice light regimes (Fig. S2).


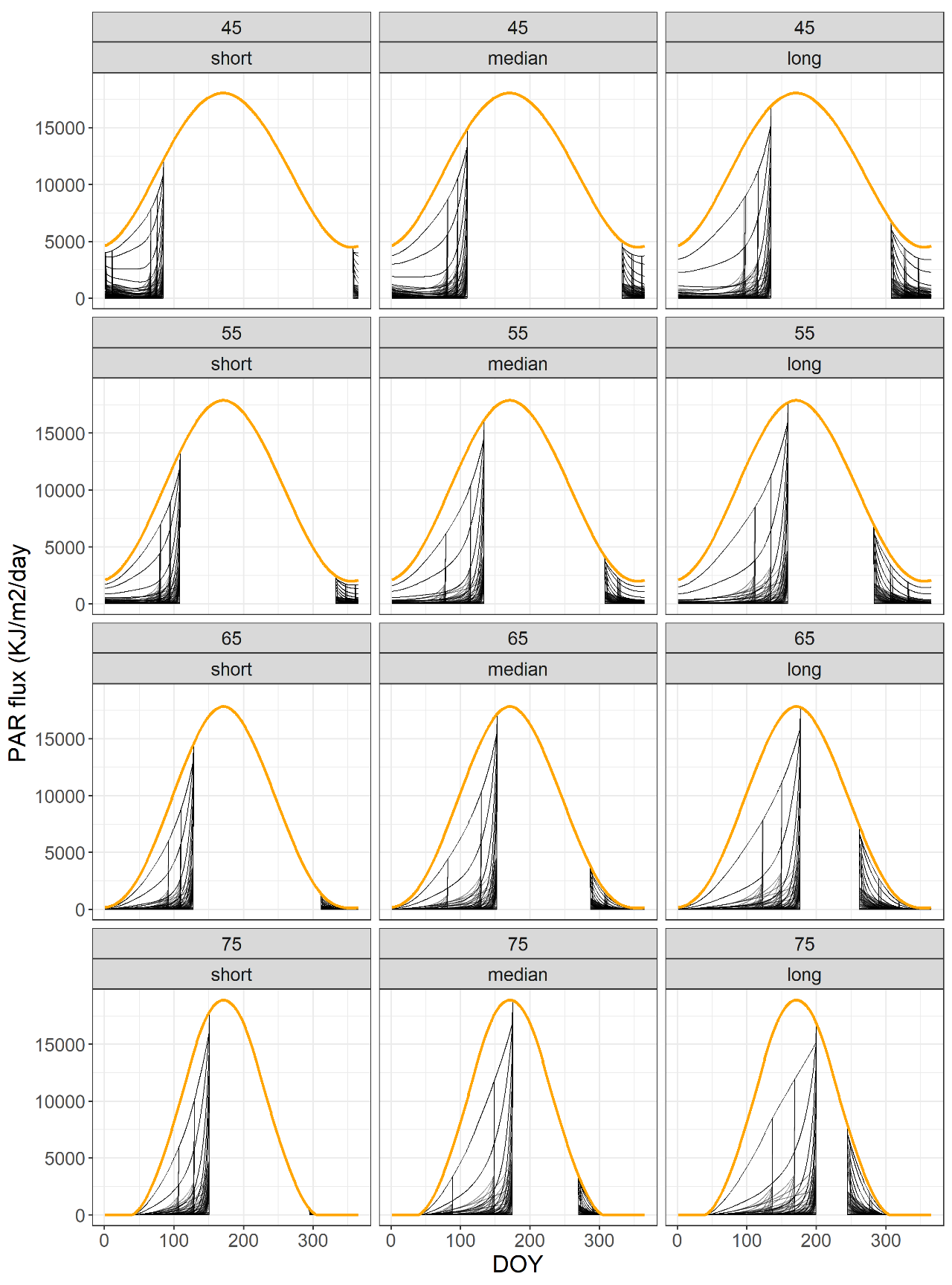


**Fig S2**: Modelled light flux scenarios. Yellow lines represent maximum (cloud-free) potential daily light flux, not accounting for attenuation by snow and ice. Thin black lines represent underice light, after accounting for attenuation by light and snow under different ice and snow duration and thickness scenarios and attenuation coefficients.
